# Cardiological Monitoring – A Cornerstone for Pediatric Inflammatory Multisystem Syndrome Temporally Associated with COVID-19 Outcome: A Case Report and a Review from the Literature

**DOI:** 10.2478/jccm-2022-0022

**Published:** 2022-11-12

**Authors:** Lorena Elena Melit, Oana Marginean, Tudor Fleșeriu, Alina Negrea, Maria Oana Săsăran, Simina Ghiraghosian-Rusu, Andrei Călin Dragomir, Mirela Oiaga, Carmen Șuteu

**Affiliations:** 1 Discipline of Pediatrics I, George Emil Palade University of Medicine, Pharmacy, Science, and Technology of Targu Mures, Mures Romania; 2 Discipline of Infectious Disease I, Mures County Hospital, Targu Mures, Mures Romania; 3 Pediatrics Clinic, Emergency County Hospital Targu-Mures, Mures Romania; 4 Discipline of Pediatrics III, George Emil Palade University of Medicine, Pharmacy, Science, and Technology of Targu Mures, Mures Romania; 5 George Emil Palade University of Medicine, Pharmacy, Science, and Technology of Targu Mures, Mures Romania; 6 Emergency County Hospital Targu Mures, Mures Romania; 7 Pediatrics Cardiology Clinic, Institute of Cardiovascular Diseases Targu Mures, Mures Romania

**Keywords:** PIMS-TS, echocardiography, systolic disfunction, outcome, child

## Abstract

**Introduction:**

Pediatric inflammatory multisystem syndrome temporally associated with COVID-19 (PIMS-TS) is a rare life-threatening condition requiring a complex management and multidisciplinary approach, whose outcome depends on the early diagnosis.

**Case report:**

We report the case of a 2 years and-5-month-old boy admitted in our clinic for fever, abdominal pain and diarrhea. The clinical exam at the time of admission revealed influenced gen-eral status, bilateral palpebral edema and conjunctivitis, mucocutaneous signs of dehydration, and abdominal tenderness at palpation. The laboratory tests performed pointed out lymphopenia, thrombocytopenia, anemia, elevated C-reactive protein – CRP, erythrocyte sedimentation rate and ferritin levels, hyponatremia, hypopotassemia, hypertriglyceridemia, elevated D-dimer, in-creased troponin and NT-proBNP. The real-time polymerase chain reaction (RT-PCR) test for SARS-CoV-2 infection was negative, but the serology was positive. Thus, established the diagnosis of PIMS-TS. We initiated intravenous immunoglobulin, empirical antibiotic, anticoagulation therapy and symptomatic drugs. Nevertheless, the clinical course and laboratory parameters worsened, and the 2nd echocardiography pointed out minimal pericardial effusion, slight dilation of the left cavities, dyskinesia of the inferior and septal basal segments of the left ventricle (LV), and LV systolic dysfunction. Therefore, we associated intravenous methylprednisolone, angiotensin converting enzyme inhibitors, spironolactone and hydrochlorothiazide, with outstanding favorable evolution.

**Conclusions:**

Echocardiographic monitoring might be a lifesaving diagnostic tool in the management of PIMS-TS.

## Introduction

The COVID-19 pandemic caused by severe acute respiratory syndrome coronavirus 2 (SARS-CoV-2) emerged in December 2019 in Wuhan, China and rapidly became a global burden being associated with severely increased morbidity and mortality rates. The worldwide reports revealed that children seem to be spared in terms of both frequency and illness severity taking into account the significantly lower incidence and admission rate as compared to adults. Nevertheless, once the pandemics evolved, several countries [1, 2, 3] reported a new pediatric inflammatory multisystem syndrome expressing a life-threatening course in a small percentage of children, which was noticed to share features with other systemic inflammatory conditions, such as Kawasaki disease, toxic shock syndrome, macrophagic activation syndrome or hemophagocytic lymphohistiocytosis [4] 2020, clinicians in the UK observed a cluster of children with unexplained inflammation requiring admission to paediatric intensive care units (PICUs. Thus, usually these children require pediatric intensive care admission if not diagnosed as early as possible and the multidisciplinary approach is mandatory in all these patients. Multiple definitions in terms of this inflammatory syndrome associated to SARS-CoV-2 infection were published by the most important health authorities worldwide (Table 1): The UK Royal College of Pediatric and Child Health (RCPCH) who named this clinical entity as pediatric inflammatory multisystem syndrome temporally associated with COVID-19 (PIMS-TS) [5], the US Center for Disease Control and Prevention (CDC)[6] and the World Health Organization [7], both referring to this syndrome as multisystem inflammatory syndrome in children (MIS-C). The lack of consensus regarding the defining criteria results mainly from the novelty of this condition, the small number of cases reported and the unexplained etiology. In spite of the differences between the three definitions, they all agree on the presence of fever in a patient with evidence of COVID-19 (previous contact with COVID-19 patients, positive real-time polymerase chain reaction for SARS-CoV-2 infection, antigen test or serology) associated with single or multi-organ dysfunction and elevated inflammatory biomarkers in the lack of other obvious microbial or viral causes [5, 6, 7]. The anamnesis is a key factor for the detection of a previous exposure to SARS-CoV-2 infection [8]equally divided into 4 groups: 25 children, 25 pediatricians, 25 care-givers (parents, tutors, and relatives, which is relevant if occurred within approximately 4 weeks prior to the onset of symptoms suggestive for PIMS-TS [6]. The awareness of this condition among parents and pediatricians is critical for the outcome of children who develop PIMS-TS taking into account the associated life-threatening complications, among which cardiac impairment is probably the most unfortunate.

**Table I:** Proposed definitions for the hyperinflammatory multisystem syndrome associated to COVID 19 in children (4-6)

Royal College of Pediatric and Child Health	Centers for Disease Control	World Health Organization
Persistent fever	Age <21 years with fever (≥38.0°C for ≥24 hours or posi- tive anamnesis for fever lasting ≥24 hours)	Age 0-19 years Fever >3 days
Inflammation (neutrophilia, elevated CRP and lymphopenia) + evidence of a single or multi-organ dysfunction	Inflammation and evidence of clinically severe illness requiring admission + multi- system (≥2) organ involvement (cardiac, gastrointestinal, renal, hematologic, dermatologic or neurologic)	AND 2 of the following: - acute gastrointestinal symptoms (vomiting, diarrhea or abdominal pain) - rash or bilateral non-purulent conjunctivitis or both (oral, hands and feet signs) - echocardiographic signs of myocardial dysfunction, pericarditis, valvulitis or coronary abnormalities, or increased troponin/NT proBNP - evidence of coagulopathy (elevated D-dimer, impaired PT or PTT
Children fulfilling complete or partial criteria for Kawasaki disease	Laboratory findings – one or more of the following: elevated CRP, ESR, fibrinogen, procalci- tonin, D-dimer, LDH, ferritin, interleukin 6, neutrophilia, lymphopenia and hypoalbuminemia	AND Elevated inflammatory biomarkers (ESR, CRP or procalcitonin)
Exclusion of other microbial cause (bac- terial sepsis, enterovirus infection asso- ciated with myocarditis, staphylococcal or streptococcal shock syndromes)	No proof of plausible alterna- tive diagnoses	AND No other identifiable microbial cause
RT-PCR for SARS-CoV-2 infection posi- tive/negative	COVID-19 exposure within 4 weeks before the onset of symptoms Positive RT-PCR, antigen test or serology for SARS-CoV-2 infection	AND Possible contact with COVID-19 patients OR Positive RT-PCR, antigen test or serology for SARS-CoV-2 infection

Legend: CRP – C-reactive protein, ESR – erythrocyte sedimentation rate, LDH – lactate dehydrogenase, RT-PCR – real time polymerase chain reaction

In terms of cardiac impairment, several echocardiographic findings are suggestive for PIMS-TS such as myocardial dysfunction, pericarditis, valvulitis or coronary abnormalities, but elevated troponin or NT-proBNP might also be encountered in patients with PIMS-TS and cardiac involvement [5, 6, 7]. Therefore, echocardiographic monitoring might be a lifesaving diagnostic tool in the management of PIMS-TS.

The aim of this case report was to increase awareness regarding cardiac involvement in children with PIMS-TS and to underline the importance of echocardiographic monitoring in the clinical course of this condition.

The informed signed consent was obtained from the patient’s mother prior to the publication of this case report.

## Case report

### Presenting concerns

We report the case of a 2 years and-5-month-old boy admitted in our clinic for fever with the onset 3 days before admission associating abdominal pain and diarrhea within the last 24 hours. The anamnesis did not reveal any possible exposure to COVID-19 infections or other chronic underlying pathologies.

### Clinical findings

The clinical exam at the time of admission revealed influenced general status, bilateral palpebral edema and conjunctivitis, mucocutaneous signs of dehydration, abdominal tenderness at palpation. The patient weighed 15 kilograms.

### Diagnostic focus and assessment

The laboratory test performed on the day of admission pointed out lymphopenia (1560/mm^3^), thrombocytopenia (108x103/mm^3^), anemia (hemoglobin – Hb 10.9 g/dL), elevated C-reactive protein – CRP (162 mg/L), erythrocyte sedimentation rate (50 mm/h) and ferritin levels (389.3 ng/mL), hyponatremia (132 mmol/ L), hypopotassemia (3.94 mmol/L), hypertriglyceridemia (476.3 mg/dL), elevated D-dimer (>5 μg/ml), increased troponin (16.6 ng/L) and NT-proBNP (19,831 pg/ml). The rapid urine exam was negative. The chest X-ray, abdominal ultrasound and echocardiography showed no pathological findings. The blood and urine culture were negative. Corroborating the previously mentioned findings we raised the suspicion of pediatric inflammatory multisystem syndrome possibly associated to COVID-19. We performed a RT-PCR test for SARS-CoV-2 infection, but it was negative. Nevertheless, the serology (IgG anti-SARS-CoV-2) for this infection was positive. Thus, established the diagnosis of PIMS-TS.

### Therapeutic focus and assessment

**Fig. 1. A:**
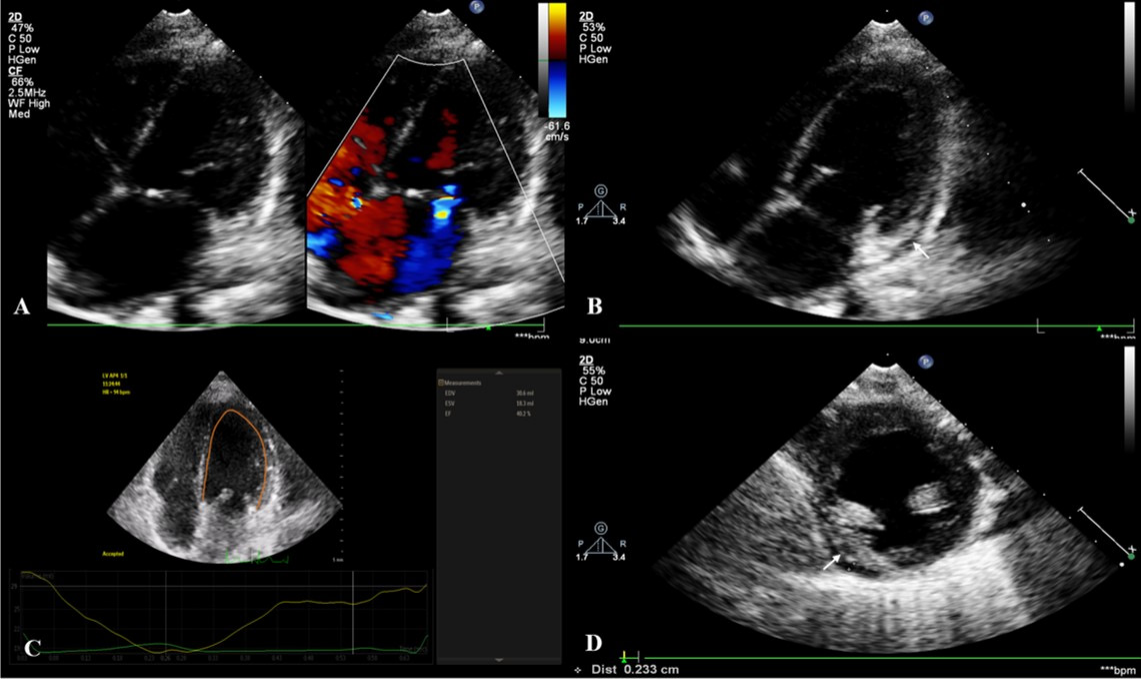
bidimensional and color Doppler echocardiography, apical four chamber view, showing dilated left cavities and mitral regurgitation; B, D: bidimensional echocardiography, four chamber and short-axis views, showing minimal pericardial effusion (arrows); C: left ventricle ejection fraction.

We initiated intravenous immunoglobulin in a dose of 2 g/kg/day associated with empirical antibiotic (ceftriaxone), anticoagulation therapy (low-molecular weight heparin) and symptomatic drugs (antipyretics, antiemetics, and antidiarrheics). In spite of this therapeutic approach, the fever persisted for the following 36 hours with no clinical improvement. Moreover, the laboratory test performed on the 3^rd^ day of admission showed an increase of the CRP value (216.65 mg/L), and the persistence of elevated NT-proBNP (9,884.2 pg/mL) and D-dimer (1,198 ng/mL), associating also hypoalbuminemia (2.45 g/dL). Therefore, we initiated intravenous methylprednisolone (10 mg/kg/day) for 3 days followed by slow tapering and substitutive treatment with human albumin. We also repeated the echocardiography and we found minimal pericardial effusion, slight dilation of the left cavities (left ventricle - LV z score: 1.6, LV sphericity index: 1.66), regional wall motion abnormalities with dyskinesia of the inferior and infero-septal basal segments of the LV. Moreover, the LV systolic dysfunction was affected, with LV ejection fraction of 40%, with mild mitral regurgitation (Figure 1); but no coronary impairment. Thus, following pediatric cardiologist’s recommendation, we associated angiotensin converting enzyme inhibitors (Lisinopril), Spironolactone and Hydrochlorothiazide. The serial echocardiography performed during the following days of admission revealed the resorption of the pericardial effusion, with improvement of LV function, correlated with decreased serum levels of NT-proBNP (123.1 pg/mL) and troponin (5.2 pg/mL).

### Follow-up and outcome

The patient was discharged on the 11^th^ day of admission without any complaints and most of the laboratory parameters within normal ranges, except of D-dimer. We recommended the continuation of anticoagulant therapy for another week, as well as Lisinopril, Spironolactone and Hydrochlorothiazide for the following 2 weeks.

## Discussions

PIMS-TS is a rare condition, but at the same time a life-threatening one requiring a complex management and multidisciplinary approach, whose outcome depends on the early diagnosis. The overlap between PIMS-TS clinical features and other inflammatory conditions represents a tricky issue in clinical practice expressing an additional burden on pediatricians facing a child with fever, which is a mandatory criterion for defining this syndrome. According to the cases reported in several countries, fever usually lasts more than 3 days [7]. Nevertheless, according to a cohort of 10 children from Italy it might last up to 6 days or more [9]. In our case the fever lasted approximately 5 days and it ceased once we introduced the intravenous treatment with methylprednisolone. Gastrointestinal symptoms like abdominal pain, vomiting or diarrhea are common symptoms in children with PIMS-TS, occurring in up to 80% of the cases [10]. Thus, abdominal pain associated with fever in pediatric patient are not uncommon signs of PIMS-TS and their importance should be overemphasized during this pandemic. Similarly, our patient also presented with fever, abdominal pain and diarrhea. Mucocutaneous changes such as rash, conjunctivitis, periorbital and peripheral edema might also be encountered in patients with PIMS-TS and they result most commonly in a misdiagnosis of Kawasaki disease [11]. Our patient presented only periorbital edema and conjunctivitis. Meningeal signs, shock, heart failure sings or thrombosis might also be present as initial signs in patients with PIMS-TS [11].

In terms of laboratory parameters, children with PIMS-TS usually presents significantly increased levels of acute phase reactants, lymphopenia, neutrophilia, elevated D-dimer and ferritin [9,11, 12, 13]. Additionally, anemia, hypoalbuminemia, elevated lactate dehydrogenase, hypertriglyceridemia associated with increased troponin and NT-proBNP in the setting of myocardial impairment might also be listed as suggestive for PIMS-TS in selected cases [9,11]. Similarly, in our case we found severely elevated CRP and ESR, lymphopenia, anemia, increased D-dimer and ferritin, hypertriglyceridemia, but also thrombocytopenia. Moreover, the laboratory test revealed in our case abnormal levels of troponin along with significantly increased NT-proBNP suggestive for cardiac impairment. PIMS-TS is definitely an immune-mediated condition since almost two-thirds of the patients are SARS-CoV-2 RT-PCR negative being usually encountered with positive serology for this condition [11,14]. Similarly, the case reported above was also identified with negative RT-PCR and positive serology. We performed a single PCR test since based on the diagnostic criteria for PIMS-TS requires either a positive PCR or positive serology.

Acute myocardial injury associated with SARS-CoV-2 infection is not an uncommon feature and it has been described both in the adult population and in children. Cardiac manifestations, including myocardial and coronary involvement, are common in children with PIMS-TS [15]. Thus, ventricular dysfunction has been reported in 35-100% of these children [15]. Elevated troponin and NT-proBNP levels have been shown to be useful markers for myocardial involvement. The underlying mechanism of myocardial injury in multisystem inflammatory syndrome in children has not been yet elucidated. Some authors, observing the clinical presentation with prolonged fever associated with severe systemic inflammation, vasoplegia, myocardial involvement and atypical form of Kawasaki disease, consider that myocardial injury is a consequence of vasculitis and inflammatory diseases following SARS-CoV-2 infection rather than direct viral organ damage [16]. Belhadjer Z et al., noting the rapid resolution of systolic LV dysfunction, together with mild to moderate troponin elevation, suggested that the mechanism of heart failure is not consistent with myocardial damage associated with acute infection with SARS-CoV-2 as seen in adults [10]. Grimaud M et al. support the hypothesis of a SARS-CoV-2 post-infective disease, noticing the cardiac function improvement as well as the significant decrease of inflammatory bio-markers following intravenous immunoglobulin[16]. These findings are further sustained by a study on French children, who were identified with significant left ventricular dysfunction and a decreased ejection fraction, which completely disappeared in 71% of the cases suggesting that myocardial edema is most-likely responsible for these injuries and not necrosis as seen in adults [10]. Similarly, our patient also developed pericarditis and left ventricular dysfunction with lower ejection fraction requiring treat, which considerably improved after approximately 10 days of anti-inflammatory and immunomodulatory therapy. Moreover, we noticed a significant decrease of both myocardial injury markers, troponin and NT-proBNP which additionally proved the improvement in cardiac function. Taking into account that multiple studies underlined that myocardial dysfunction occurs in approximately 60% of the children with PIMS-TS, echocardiography is extremely important for the proper diagnosis of this complex condition. Moreover, in our case the initial echocardiography was normal suggesting that myocardial dysfunction might also occur during the clinical course of this condition. Therefore, serial echocardiography might considerably improve the outcome of these children.

Intravenous immunoglobulin was reported as first line therapy in children with PIMS-TS due to the similarities with Kawasaki disease since it might favor the neutralization of virus and related superantigens down-regulating the cytokine storm [11,17,18]. Nevertheless, as it was also seen in patients with Kawasaki disease [19,20], certain cases do not respond to this therapy, and corticosteroids were proved to be useful adjuvants in this subgroup [9,11,14]. Thus, the use of an initial pulse intravenous methylprednisolone regime (10 mg/ kg/day for 3 days) followed by a progressive tapering regimen might decrease the length of fever, being associated with a lower risk of cardiac impairment [11,19]. Taking into account the cytokine elevation in PIMS-TS, biological treatment might represent an extreme alternative when the fever and inflammation persist despite intravenous immunoglobulin and corticosteroids [21,22].

## Conclusions

PIMS-TS is a novel condition and much has to be learnt about it among the health care providers. Nevertheless, its early diagnosis is crucial and increased awareness among both pediatricians and parents represents the key to the best outcome. Echocardiography monitoring is mandatory during the clinical course of PIMS since cardiac involvement might occur at any time during the evolution. It is true that we are novices in terms of PIMS and the path of its understanding is burdened by a wide-spectrum of unknown variables which can only be solved by corroborating all the valuable experience in this field.
